# Differential Expression of Peroxisomal Proteins in Distinct Types of Parotid Gland Tumors

**DOI:** 10.3390/ijms22157872

**Published:** 2021-07-23

**Authors:** Malin Tordis Meyer, Christoph Watermann, Thomas Dreyer, Steffen Wagner, Claus Wittekindt, Jens Peter Klussmann, Süleyman Ergün, Eveline Baumgart-Vogt, Srikanth Karnati

**Affiliations:** 1 Department of Otorhinolaryngology, Head and Neck Surgery, University of Giessen, Klinikstrasse 33, Ebene-1, D-35392 Gießen, Germany; Meyer@jokba.de (M.T.M.); Christoph.Watermann@chiru.med.uni-giessen.de (C.W.); Steffen.Wagner@hno.med.uni-giessen.de (S.W.); Claus.Wittekindt@klinikumdo.de (C.W.); jens.klussmann@uk-koeln.de (J.P.K.); 2 Institute of Pathology, Justus Liebig University, Langhansstrasse 10, D-35392 Gießen, Germany; th.dreyer@gmx.de; 3 Department of Otorhinolaryngology, Head and Neck Surgery, Medical Faculty, University of Cologne, D-50931 Cologne, Germany; 4 Institute for Anatomy and Cell Biology, Julius-Maximilians-University Würzburg, Koellikerstrasse 6, D-97070 Würzburg, Germany; sueleyman.erguen@uni-wuerzburg.de; 5 Institute for Anatomy and Cell Biology II, Medical Cell Biology, Justus Liebig University, D-35385 Gießen, Germany; eveline.baumgart-vogt@anatomie.med.uni-giessen.de

**Keywords:** peroxisomes, parotid gland, salivary, tumors, pleomorphic adenoma, mucoepidermoid carcinoma, acinic cell carcinoma, differential expression, immunohistochemistry, mRNA

## Abstract

Salivary gland cancers are rare but aggressive tumors that have poor prognosis and lack effective cure. Of those, parotid tumors constitute the majority. Functioning as metabolic machinery contributing to cellular redox balance, peroxisomes have emerged as crucial players in tumorigenesis. Studies on murine and human cells have examined the role of peroxisomes in carcinogenesis with conflicting results. These studies either examined the consequences of altered peroxisomal proliferators or compared their expression in healthy and neoplastic tissues. None, however, examined such differences exclusively in human parotid tissue or extended comparison to peroxisomal proteins and their associated gene expressions. Therefore, we examined differences in peroxisomal dynamics in parotid tumors of different morphologies. Using immunofluorescence and quantitative PCR, we compared the expression levels of key peroxisomal enzymes and proliferators in healthy and neoplastic parotid tissue samples. Three parotid tumor subtypes were examined: pleomorphic adenoma, mucoepidermoid carcinoma and acinic cell carcinoma. We observed higher expression of peroxisomal matrix proteins in neoplastic samples with exceptional down regulation of certain enzymes; however, the degree of expression varied between tumor subtypes. Our findings confirm previous experimental results on other organ tissues and suggest peroxisomes as possible therapeutic targets or markers in all or certain subtypes of parotid neoplasms.

## 1. Introduction

Salivary gland cancers are rare neoplasms accounting for 3–6% of all head and neck cancers with an annual incidence of 16/1,000,000 population, a 5-year survival rate of 95% if confined to gland and a 44% survival rate in the case of distant metastasis [1,2,3]. In this case, 80% of those tumors arise in the parotid gland, 10–15% arising in the submandibular gland, and the remainder arising in sublingual and minor salivary glands [4]. Owing to the mixed composition of epithelial and non-epithelial tissue in affected salivary glands, up to 33 histological subtypes of parotid tumors are possible, leading to the variable clinical behavior of neoplasms [5]. The most frequent benign salivary gland tumor is pleomorphic adenoma (PMA). Common histopathological types are mucoepidermoid carcinoma (MEC) and acinic cell carcinoma (ACC) [6]. We focused our experiments on deciphering peroxisome characteristics in the above three prevalent parotid gland histopathological tumor types.

Peroxisomes are subcellular, single membrane-bound and morphologically heterogeneous eukaryotic structures characterized by a homogenous, granular matrix rich in concentrated and diverse proteomes [7,8]. They contribute an important part to cell metabolism and are responsible for divergent biochemical anabolic and catabolic processes including ether phospholipid biosynthesis as well as the α- and β-oxidation of fatty acids, which is essential for the integrity of cell membranes [9,10]. Peroxisomes also catalyze a multitude of key processes, such as scavenging bioactive hydrogen peroxide, redox homeostasis, lipidic intra- and inter-cell signaling and cellular transportation [11,12]. Furthermore, peroxisomes regulate early embryonic development as well as cellular differentiation and survival and were recently shown to modulate the innate immune system [13,14,15]. Despite this, many of physiological roles of peroxisomes remain enigmatic [16]. 

The efficient functioning and co-ordination of peroxisomes relies on peroxisome machinery. This entails the regulation of peroxisomal proliferation, maintenance, pexophagy (degradation) and compartmentalization of matrix proteins by an array of specialized proteins [17]. These constitute at least 130 different peroxisomal proteins, and their abundance is specific to cell type, metabolic demand and tissue microenvironment [18]. These peroxisomal proteins include: oxidases for the β-oxidation of fatty acids, such as D-amino acid oxidase, acyl-coA oxidase and acyl-coA synthetase; catalases for hydrogen peroxide scavenging; HMG-CoA reductase for cholesterol synthesis; ATP Binding Cassette subfamily D (ABCD), responsible for transporting matrix proteins; 14 peroxins, such as PEX19, PEX3 and PEX16; and peroxisome-proliferator-activated receptors (PPARs) with their three identified isotopes—PPARα, PPARβ/δ and PPARγ—which are involved in peroxisome proliferation, membrane transport and macropexophagy via regulation of peroxisomal genes [19]. 

Peroxisomes are central to pathways that are paramount for healthy living cells; they are critical for normal human health and development. It is not surprising, therefore, that disorders of peroxisomes have proven to contribute to the pathophysiology of human neurodegenerative diseases that are attributed fundamentally to altered anti-oxidative stress, such as diabetes, obesity, aging and age-related disorders [20,21]. With the advent of high-throughput next-generation genetic sequencing and advances in molecular approaches, it has been demonstrated that cancer has aberrant metabolism compared to healthy tissue [22]. Subsequently, deregulation of peroxisome dynamics has been hypothesized for its pro-tumorigenic role [23,24]. The earliest plausible peroxisomal role in carcinogenesis was examined in human breast, liver and colonic carcinoma specimens in the 1990s. In these studies, a downregulation of peroxisomal enzymes was found [25,26,27]. No significant difference was noted between numbers of peroxisomes in normal and tumoral breast epithelia; however, catalase-positive organelles and enzyme activity was found to be less numerous and lower in neoplastic colonocytes [28]. Recent data has indicated that enzymes involved in peroxisomal lipid processing are elevated in many tumor types including prostate cancer [29], colorectal carcinomas [30], as well as breast, ovarian and bladder cancer [31]. Intriguingly, it was observed that the activity of specific peroxisomal enzymes correlate with the histopathological grade of the tumor, suggesting peroxisomes as one means of tumor grading [32]. On the other hand, depletion of PEX2 was lethal to hepatic cell carcinoma in a xenograft mouse model via inhibiting the mTOR pathway, a common signaling pathway involved in proliferation and survival of tumor tissue, thus suggesting a context or tissue-specific response to peroxisomal deregulation [33]. However, there is still a significant gap in our knowledge of how peroxisomes influence pro- or anti-tumor metabolism. To the best of our knowledge, there are no studies that either compare peroxisomal dynamics in normal human parotid salivary gland specimens against their neoplastic counterparts or the differences across its common clinical histopathological subtypes (PMA, MEC and ACC). Therefore, we attempt to use immunofluorescence (IF) tissue staining and quantitative polymerase chain reaction (qPCR) techniques to ascertain if significant differences of peroxisomal proteins can be observed at a morphological and or molecular biological level between healthy and neoplastic cells of the human parotid salivary gland.

## 2. Results

### 2.1. Pathology of Parotid Tumor Entities Using HE Staining 

**Figure 1:**
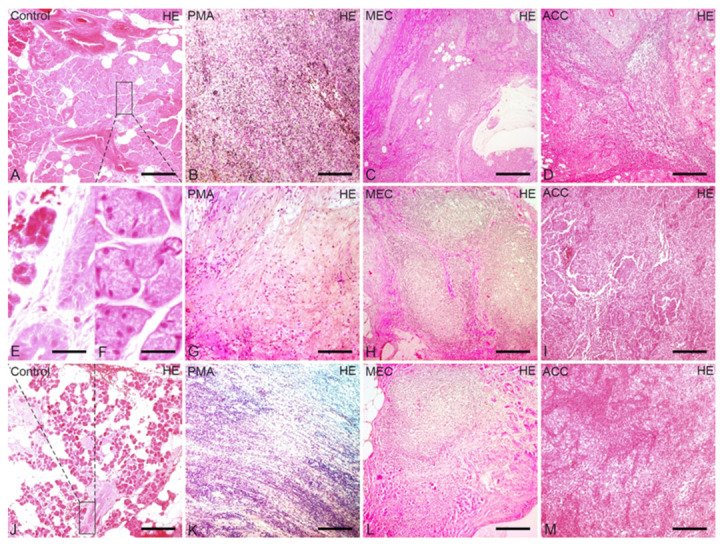
Comparison of the structural differences between control, PMA, MEC and ACC parotid gland tissue using HE staining: Control parotid gland 1 in HE staining (**A**) and the magnification of its acinic cells in HE staining (**F**). Control parotid gland 2 in HE staining (**J**) and the magnification of its striated duct cells in HE staining (**E**). In comparison, MEC (**C**,**H**,**L**) consists mostly of squamous and mucus-forming epithelium and ACC (**D**,**I**,**M**) consists of uniform acinar cells. In contrast, PMA (**B**,**G**,**K**) consists of epithelial and mesenchymal lineage differentiations. Bars represent 175 µm: (**A**–**D**,**G**–**M**); 32, 5 µm: (**E**,**F**).

Using conventional HE staining, resected normal parotid gland tissue specimens appeared as parenchymal (secretory) tissue divided into lobules by the stromal fibrous connective tissue septa in which large excretory interlobular ducts were seen under lower magnification (Figure 1A,J). Under high magnification, 5–7 round to oval clusters of darkly stained uniform-sized basophilic glandular cells with round basal nuclei and indistinct borders appeared, surrounded by few flat-shaped myoepithelial cells characteristic of normal serous acinar cells. These acinar cells were admixed with few adipocytes and surrounding cuboidal epithelium-lined intra-lobular striated ducts (Figure 1E,F). In PMA, the normal architecture, as outlined, was partly replaced by pseudo-capsular pleomorphic sheets of duct-like structures, clumps and/or interlacing strands of polygonal or stellate-shaped myo-epithelial cells, but some normal acinar structures are still visible (Figure 1B,G,K). MEC appeared as non-capsulated solid sheets of epidermoid polygonal cell nests with areas of necrosis and clear cell changes in the form of glycogen accumulation (Figure 1C,H,L). ACC showed biphasic neoplastic tissue composed of a mixture of cord-like or trabecular glandular cells and basaloid myoepithelial cells with dark angulated nuclei and limited cytoplasm (Figure 1D,I,M).

### 2.2. Myoepithelial and Acinar Cell Markers in Parotid Tumor Entities

**Figure 2:**
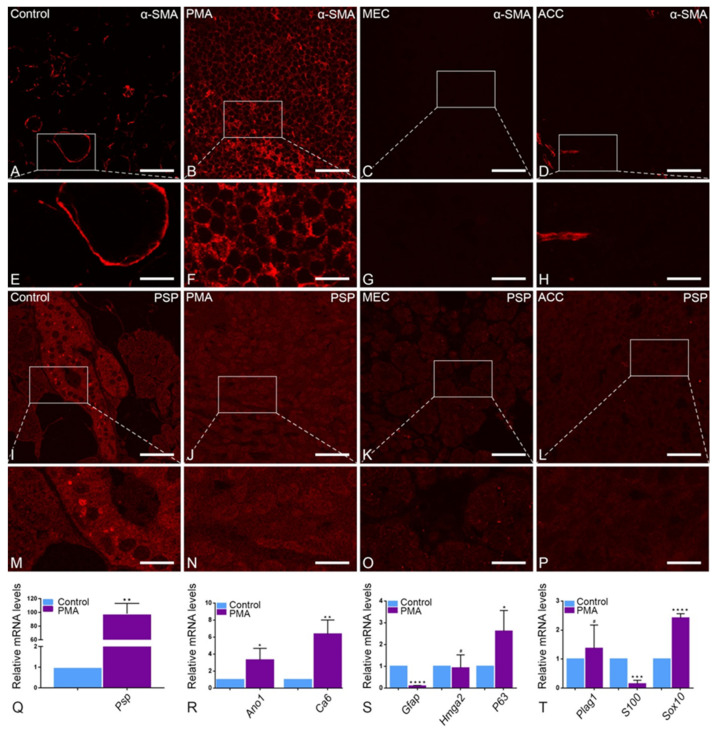
Comparison of control, PMA, MEC and ACC parotid gland tissue in αSMA and PSP staining: Control parotid gland in αSMA staining (**A**) reacts positive as well as PMA in αSMA staining (**B**), and their respective magnifications-control (**E**) and PMA (**F**). In αSMA staining of MEC (**C**) and its magnification (**G**), just as ACC (**D**) and its magnification (**H**), shows significantly less reaction. Control parotid gland (**I**) shows a high abundance of PSP, especially in the magnification of its striated ducts (**M**). PMA (**J**) and its magnification (**N**) react in a similar pattern to the control group, but with a slightly higher intensity. PSP-stained MEC (**K**), its magnification (**O**) and PSP-stained ACC (**L**), and its magnification (**P**), show weaker responses compared to the control group. mRNA expression of Psp in control parotid gland and PMA as determined by real-time PCR analysis (**Q**) quantify the results of immunofluorescence (** *p* = 0.0030). The mRNA expression of Ano1 (* *p* = 0.0420) and Ca6 (** *p* = 0.0049) (**R**), P63 (* *p* = 0.0426) (**S**), as well as Plag1 and Sox10 (**** *p* < 0.0001) (**T**) in the control parotid gland and PMA as determined by real-time PCR analysis show a higher abundance in PMA. In comparison, the mRNA levels of Gfap (**** *p* < 0.0001), Hmga2 (**S**) and S100 (*** *p* = 0.0003) (**T**) are expressed lower in PMA. Bars represent 50 µm: (**A**–**D**,**I**–**L**); 18 µm: (**E**–**H**,**M**–**P**). (Explanation of the notations: The number of asterisks signifies the degree of significance of the *p*-value, while # means not significant.)

IF staining showed the periductal cytoplasmic staining pattern of αSMA heavy chains, a marker of myoepithelial cells, around striated ducts in healthy tissue (Figure 2A,E) compared to a higher intensity staining around residual acinar and ductal structures in PMA, correlating with the predominant secretory nature of those tumors (Figure 2B,F). MEC specimens showed classic negative staining for αSMA (Figure 2C,G), while ACC showed scant, patchy positive staining for αSMA, correlating with islands of activated myofibroblasts (Figure 2D,H). The parotid specific protein (PSP) antibody showed a specific positive staining of the secretory granules at ultrastructural level and is highly abundant in the human parotid gland [34]. Therefore, we used it as a parotid specific marker protein to identify the correct tissue before characterizing the peroxisomal compartment. Indeed, the labeling of PSP showed a positive staining of the secretory granules (Figure 2I,M) in the control tissue, suggesting the isolated tissue was a typical parotid gland. Higher magnification of the acini showed many big and spherical secretory granules (Figure 2M). However, in tumor entities PSP labeling was significantly less abundant compared to the control tissue (Figure 2I–P). Surprisingly, analysis of mRNA expression levels for PSP did not corroborate with the morphological findings. The Psp mRNA showed a significantly higher expression in the PMA in comparison to the human parotid gland (Figure 2Q). To ascertain the PMA tumor tissue that was used for experiments, we performed a gene expression profile of possible markers, which were used to differentiate between the different entities. In PMA, compared to healthy tissue, quantitative-PCR showed classic differential mRNA over-expression of carbonic anhydrase 6 (Ca6) and anoctamin 1 (Ano1), markers of differentiated serous acinar cells; P63, a myoepithelial marker; and Sox10, a non-specific marker of salivary glandular tissue. Striking over-expression of pleomorphic adenoma gene 1 (Plag1), a proto-oncogene, was detected in PMA compared to healthy tissue. In contrast, the mRNAs encoding for S100 and Gfap were significantly downregulated in PMA tumors in comparison to control tissue (Figure 2S,T).

### 2.3. Differential Expression of Peroxisomal Biogenesis, Matrix Proteins and Enzymes in Parotid Tumors

#### 2.3.1. Peroxisomal Biogenesis Proteins

**Figure 3:**
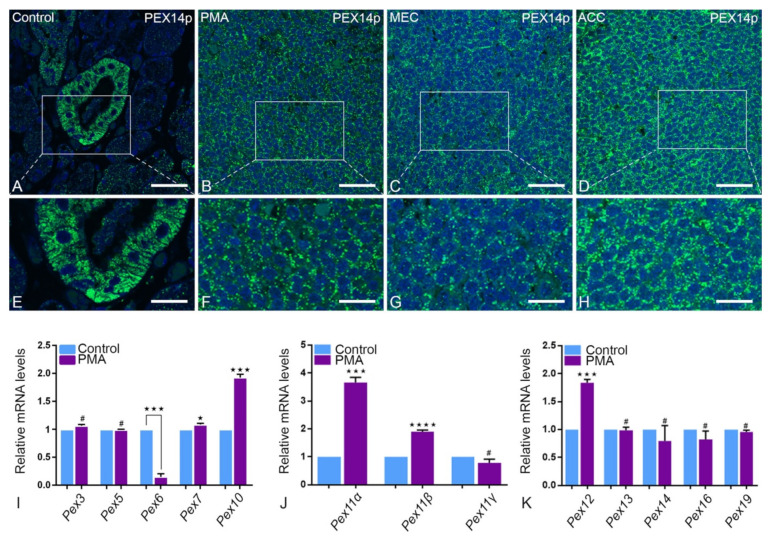
Peroxisomal biogenesis proteins and mRNAs in PMA, MEC and ACC: Control parotid gland PEX14p staining (**A**) is evenly distributed among the acinar cells. The striated duct cells in the magnification (**E**) show a high expression of PEX14p. In comparison PMA (**B**), MEC (**C**) and their respective magnifications (**F**,**G**) show a lower reaction level than the duct cells and the acinar cells in relation to the cell number. In ACC (**D**,**H**), a higher abundance of PEX14p can be detected compared to the two other tumor types. This is confirmed by the mRNA expression of Pex14 in PMA (**K**). Some Pex genes are expressed lower in PMA than in the control group, such as Pex6 (*** *p* = 0.0001) in (**I**), Pex11γ in (**J**), as well as Pex16 and Pex19 in (**K**). Only in the first case is the down regulation significant. In contrast, Pex7 (* *p* 0.0178) and Pex10 (*** *p* = 0.0001) in (**I**), Pex11 α (*** *p* = 0.0001) and β (**** *p* < 0.0001) in (**J**) and Pex12 (*** *p* = 0.0002) in (**K**) are significantly upregulated. Bars represent 50 µm: (**A**–**D**); 18 µm: (**E**–**H**). (Explanation of the notations: The number of asterices signifies the degree of significance of the *p*-value, while # means not significant.)

As shown by IF analysis, control parotid tissue showed the typical punctate staining pattern of peroxisomal PEX14p, an established marker protein used to visualize the abundance of peroxisomes, with high fluorescence corresponding to peroxisome membranes. PEX14p intense IF staining was detected in the striated ducts of the control tissue only. In tumorous tissues, PEX14p IF staining was less abundant suggesting less peroxisome biogenesis (Figure 3A–H). Further, qRT-PCR analysis of mRNA encoding for peroxisomal biogenesis protein 6 (Pex6) showed significantly lower expression in PMA in comparison to healthy tissue. In contrast, analysis for mRNAs encoding for peroxisomal biogenesis proteins (Pex7, Pex10, Pex12), and proteins for peroxisomal proliferation (Pex11α, Pex11β) were significantly upregulated in PMA tumor compared to control tissue (Figure 3J,K).

#### 2.3.2. Peroxisomal β-Oxidation Enzymes of Pathway 1 and 2

**Figure 4:**
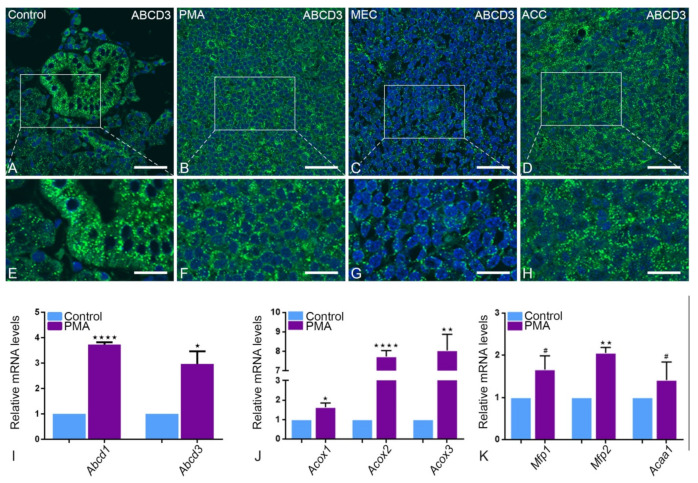
Peroxisomal proteins and mRNAs involved in lipid transport and β-oxidation pathways: ABCD3 is more highly expressed in striated duct cells (**E**) than in acinar cells (**A**). In comparison, the marker reacts low in MEC (**C**,**G**). PMA (**B**,**F**) and ACC (**D**,**H**) show an even higher expression in IF than the control group. This is validated by the higher mRNA expression of Abcd1 (**** *p* < 0.0001) and Abcd3 (* *p* = 0.0164) in (**I**). In addition, Acox1 (* *p* = 0.0404), Acox2 (**** *p* < 0.0001) and Acox3 (** *p* 0.0010) (**J**), Mfp1, Mfp2 (** *p* = 0.0012) (**K**) and Acaa1 show a high abundance in PMA mRNA levels. Bars represent 50 µm: (**A**–**D**); 18 µm: (**E**–**H**). (Explanation of the notations: The number of asterices signifies the degree of significance of the *p*-value, while # means not significant.)

IF showed higher expression of the distinct ATP binding cassette subfamily D3 (ABCD3), required for the transport of lipid into the peroxisomes. ABCD3 was highly expressed in PMA tumor tissue compared to healthy tissue. In contrast, in ACC and MEC, ABCD3 seems to be expressed to a lesser extent in tumor than in healthy tissue (Figure 4A–H). In qRT-PCR, the mRNA expression levels of Abcd1 and Abcd3 were higher in PMA tumor tissue compared to those in healthy tissue (Figure 4I). Interestingly, in addition to the ABCD family, all other enzymes involved in β-oxidation, namely Acox1, Acox2, Acox3, Mfp1, Mfp2 and Acaa1, showed higher mRNA expression in PMA type of parotid tissue than healthy tissues in qRT-PCR (Figure 4J,K).

#### 2.3.3. Plasmalogen and Cholesterol Synthesis Enzymes

**Figure 5:**
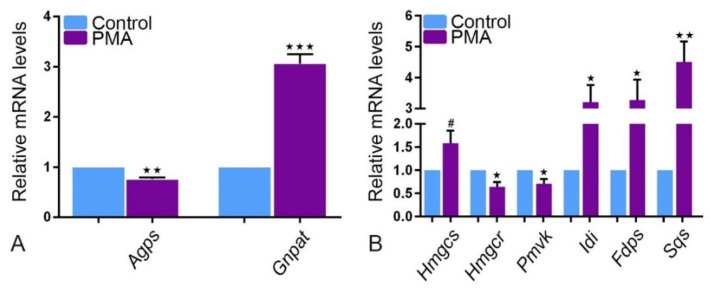
Peroxisomal ether lipid and cholesterol synthesis in PMA. The mRNA expression by real-time PCR analysis of (**A**) Agps (** *p* = 0.0018), (**B**) Hmgcr (* *p* = 0.0235) and Pmvk (* *p* = 0.0478) are expressed lower while Gnpat (*** *p* = 0.0004) (**A**), Hmgcs, Idi (* *p* = 0.0155), Fdps (* *p* = 0.0257) and Sqs (** *p* = 0.0059) (**B**) are expressed higher in PMA than in healthy tissue. (Explanation of the notations: The number of asterices signifies the degree of significance of the *p*-value, while # means not significant.)

We observed a higher expression of the glycerone-phosphate O-acyl transferase (Gnpat), an ether lipid synthesizing enzyme, in PMA tumor tissue relative to healthy tissue, but alkylglycerone phosphate synthase (Agps) was less expressed (Figure 5A). A differential mRNA expression of cholesterol synthesizing enzymes was observed. In PMA tumor tissue (Figure 5B), significant downregulation of HMG-CoA reductase (Hmgcr) and phosphomevalonate kinase (Pmvk) was detected compared to increased expression levels of mevalonate 5-disphosphate decarboxylase (Mvd), Farnesyl diphosphate synthase (Fdps), 3-hydroxy-3-methylglutaryl-CoA synthase (Hmgcs), isopentenyl diphosphate isomerase (Idi) and squalene synthase (Sqs), an mRNA encoding for ER enzyme.

#### 2.3.4. Catalase, Superoxide Dismutase and Extra-Peroxisomal Antioxidative Enzymes

**Figure 6:**
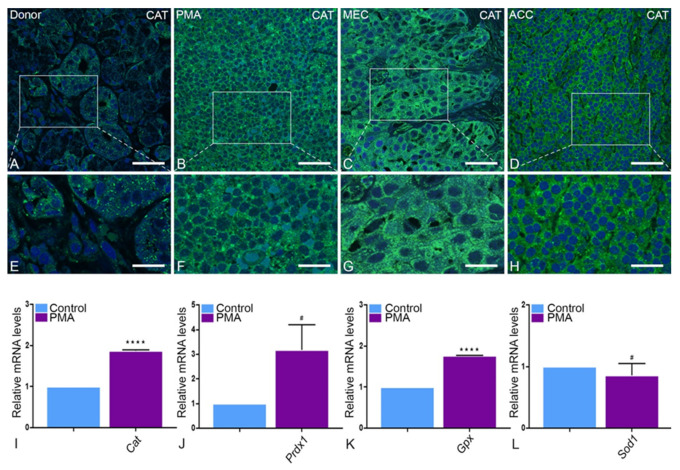
Expression of catalase in PMA, MEC and ACC: In the control parotid gland tissue and its magnification (**A**,**E**), CAT is expressed significantly lower than in PMA (**B**,**F**), MEC (**C**,**G**) and ACC (**D**,**H**). This result is validated by mRNA expression of Cat in the control parotid gland tissue and PMA (**** *p* < 0.0001) (**I**) as determined by real-time PCR analysis. The mRNA levels of Prdx1 (**J**) and Gpx (**** *p* < 0.0001) (**K**) are higher in PMA than in the control group as well. Compared with the control, only the real-time PCR mRNA levels of Sod1 (**L**) are expressed lower in PMA. Bars represent 50 µm: (**A**–**D**); 18 µm: (**E**–**H**). (Explanation of the notations: The number of asterices signifies the degree of significance of the *p*-value, while # means not significant.)

**Figure 7:**
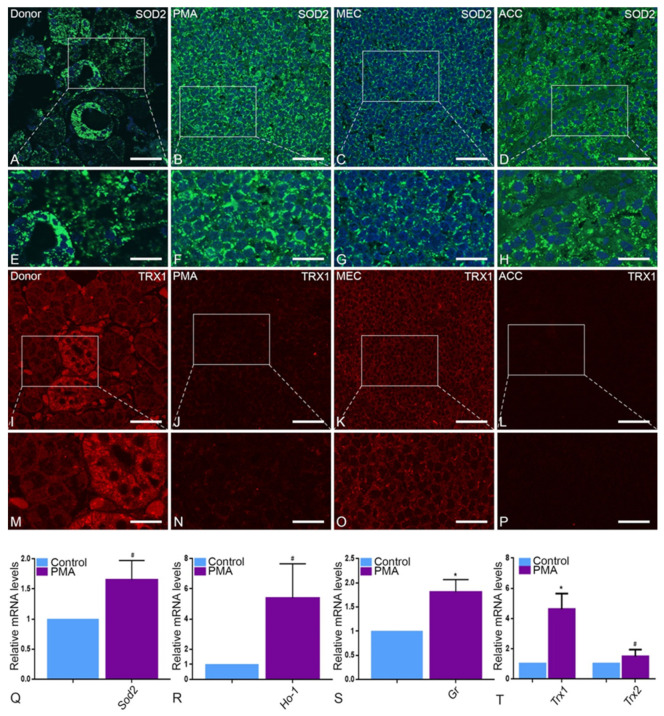
Anti-oxidative enzymes in PMA, MEC and ACC: In healthy parotid tissue (**A**,**E**), SOD2 is more abundant in striated duct than in acinar cells. SOD2 is highly expressed in PMA (**B**,**F**) and ACC (**D**,**H**). Accordingly, in real-time PCR analysis, Sod2 is significantly increased in PMA (**Q**) as well as Ho-1 (**R**) and Gr (* *p* = 0.034) (**S**). MEC (**C**,**G**) shows weak expression of SOD2. In immunofluorescence of TRX1, the control group (**I**,**M**) shows a strong expression especially in the striated duct cells. In addition, MEC (**K**,**O**) reacts positive, while PMA (**J**,**N**) and ACC (**L**,**P**) show less reaction. This contradicts the mRNA expression of Trx1 (* *p* = 0.0262) and Trx2 (**T**) by real-time PCR analysis. Bars represent 50 µm: (**A**–**D**,**I**–**L**); 18 µm: (**E**–**H**,**M**–**P**). (Explanation of the notations: The number of asterices signifies the degree of significance of the *p*-value, while # means not significant.)

Catalase exhibits a punctuate pattern in healthy tissue and is particularly abundant in striated ducts (Figure 6A,E). Since the normal tissue architecture of parotid glands is lost during neoplastic transformation (Figure 6B–D,F–H), we were only able to observe an apparently higher expression of catalase in tumor tissue without reference to a specific glandular structure. Moreover, catalase was mis-localized into cytoplasm in all tumor entities in comparison to the healthy tissue (Figure 6B–H). In qRT-PCR, catalase showed a significant upregulation in tumor tissue in comparison to control tissue (Figure 6I). It is worth noting that mRNA expression levels of other antioxidative enzymes, namely peroxiredoxin 1 (Prdx1), glutathione peroxidase (Gpx), but not superoxide dismutase 1 (Sod1), were similarly higher in the PMA type of tumor tissue than in healthy parotid tissue (Figure 6J–L). Specifically, superoxide dismutase 2 (SOD2) showed a scattered pattern in tumor tissue under IF (Figure 7B–D,F–H) as opposed to its typical mitochondrial localization in healthy tissue (Figure 7A,E). Moreover, thioredoxin reductase 1 (TRX1), an antioxidant protein enzyme ubiquitous in mammals and essential for life, showed typical IF staining in healthy tissue but an incremental decrease in intensity from PMA, to MEC and ACC accordingly. The ACC type of tumor tissue showed a complete absence of TRX1 IF staining (Figure 7I–P). In qRT-PCR, mRNA expression levels of Sod2, heme oxygenase 1 (Ho-1) and glutathione reductase (Gr) were higher in the PMA type of parotid tumor tissue (Figure 7Q–T) in comparison to healthy tissue. Surprisingly, mRNA expression analysis for Trx1 showed a significant upregulation in PMA in comparison to healthy tissue, which is not in agreement with IF staining. Moreover, TRX2 was slightly upregulated in PMA; however, this was not statistically significant (Figure 7T).

### 2.4. Peroxisome Proliferator-Activated Receptors (PPARs)

**Figure 8:**
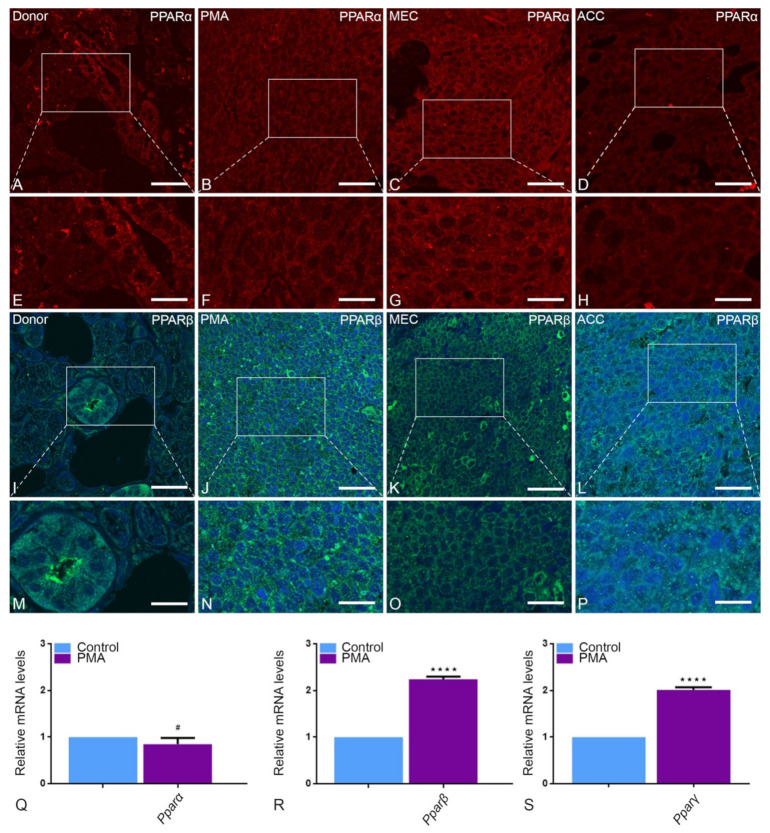
Peroxisomal proliferator-activated receptors in PMA, MEC and ACC: In healthy parotid tissue, a positive reaction of PPARα (**A**,**E**) and PPARβ (**I**,**M**) can partially be observed, especially in striated duct cells. PPARα staining is strong in PMA (**B**,**F**) and MEC (**C**,**G**) as also observed in the mRNA expression of PMA (**Q**). In contrast, ACC (**D**,**H**) shows less reaction compared to the two other tumor tissues. In IF, PPARβ is more abundant in PMA (**J**,**N**) and ACC (**L**,**P**) than in MEC (**K**,**O**). The high expression of Pparβ in PMA (**R**) can also be detected by real-time PCR analysis (**** *p* < 0.0001). Accordingly, the mRNA levels of Pparγ are abundant in PMA (**** *p* < 0.0001) (**S**). Bars represent 50 µm: (**A**–**D**,**I–L**); 18 µm: (**E**–**H**,**M**–**P**). (Explanation of the notations: The number of asterices signifies the degree of significance of the *p*-value, while # means not significant.)

PPARs regulate peroxisomal proteins by binding to promoters of peroxisomal genes. Therefore, all three PPAR family members (PPARα, -β and -γ) were characterized in parotid tumors. Interestingly, PPARα was downregulated in all tumor entities in comparison to healthy parotid tissue (Figure 8A–H). However, a clear upregulation of PPARβ was detected in PMA and in MEC while a downregulation was detected in ACC parotid tumor entities in comparison to healthy tissue (Figure 8I–P). The antibody against PPARγ did not yield any reaction. In contrast, qRT-PCR results demonstrated a significant upregulation of Pparß and -γ in neoplastic tissues compared to healthy tissue (Figure 8R, S). Further, a downregulation of Pparα was noted in PMA in comparison to healthy tissue; however, this was not statistically significant (Figure 8Q). 

## 3. Discussion

Rapidly proliferating tumor cells have their cellular metabolism reprogrammed by the direct and indirect consequences of underlying oncogenic mutations. Tumor cells with rewired metabolic pathways confer the selective advantage of enhanced influx of necessary and unconventional nutrients that are required to sustain deregulated proliferation towards creation or expansion of its biomass [11,23]. One of the many metabolic demands of a tumor cell is meeting its need for the massive amount of cell membrane production required to progress. As peroxisomes play a cornerstone role in lipid metabolism, we started our experiments by examining the gene expression of proteins that regulate peroxisomal biosynthesis, the peroxins (PEX), which are peroxisomal biogenesis factors essential in regulating peroxisomal assembly and function. To date, 34 PEX genes encoding peroxins have been identified [35,36]. Of these, at least 13 PEX genes are required for proper peroxisomal biogenesis [37]. The universal lower expression of PEX14p, a docking protein required for translocation of matrix proteins into the peroxisomes, in our tumor samples suggests an impaired peroxisomal biogenesis parotid neoplasia. Only PEX11 and PEX12 were differentially upregulated in parotid tumor tissue compared healthy parotid tissue. Downregulation of some peroxisomal biogenesis proteins, such as PEX6, alongside upregulation of others (PEX 7, 10, 11α, 11β, 12) in our PMA samples may suggest tumorigenic addiction, and thus could be targeted therapeutically. However, while the implications of upregulation of certain PEX genes compared to others in tumor parotid cells are unclear, this could indicate the presence of a deregulated peroxisomal biogenesis process. Mutations in PEX genes typically result in defective peroxisomes due to the mis-localization of peroxisomal matrix proteins to the cytosol [38]. Inherited bi-allelic mutations in PEX genes cause peroxisomal biogenesis disorders (PBD). PBDs often have severe neurological manifestations attributed to defective neuronal myelin sheath, which results from peroxisomal defects in fatty acid metabolism [39]

Peroxisomal enzymes oxidize both linear and branched forms of very long chain unsaturated fatty acids to generate fatty acyl-CoA and acetyl-CoA that substrates enter in plasma membrane biosynthesis [40]. The transport of these substrates across peroxisomal membranes is dependent on the ATP-binding cassette (ABC) transporter subfamily D (ABCD1, ABCD2, ABCD3) [41]. Functions of these subfamily members overlap; however, ABCD1 and ABCD2 preferentially transport hydrophobic saturated very long chain fatty acids, while ABCD3 (PMP70) distinctly imports hydrophilic branched chain fatty acyl-CoAs across the peroxisomal membrane [42,43]. Another family of enzymes is the FAD-dependent acyl-coA oxidases and multifunctional proteins (MFP) involved in subsequent β-oxidation of the imported acyl-coA substrates [44,45,46]. Indeed, we found a characteristically higher expression of ABCD3 in parotid tumor cells compared to their healthy counterpart, suggesting an enhanced lipid trafficking across peroxisomes. This is consistent with previous findings that dysregulation of ABC protein expression takes place in both solid and diffuse tumors [47,48]. 

Interestingly, the lower overexpression of ABCD3 in our malignant MEC and ACC tissue compared to PMA, a tumor of benign nature, may suggest a metabolic shift to alternative pathways during malignant transformation of benign parotid tumors. Thus, in parotid gland tumors, downregulation of ABCD3 transporter may confer an association with progression to a malignant phenotype such as to MEC and ACC; however, further studies remain to confirm or negate our hypothesis [49]. Moreover, the aberrant overexpression of the ACOX and MFP families of enzymes found exclusively in PMA parotid tumor cells are in concordance with a previous finding in certain subtypes of breast cancer cells [50]. However one should keep in mind that not all protein expression is attributed to correlative changes in mRNA levels [51]. 

In the same context, our parotid tumor cells showed elevated levels of lipogenic enzymes (i.e., plasmalogen and cholesterol synthesis enzymes). This is consistent with other studies suggesting that high levels of ether phospholipids in some tumor types implies that elevated peroxisomal lipid synthesis is associated with tumor progression [52].

It cannot be ignored that peroxisomal enzymes of β-oxidation yield byproducts such as hydrogen peroxide (H_2_O_2_), reactive oxygen and nitrogen species (ROS/RNS) [53]. Since these byproducts are genotoxic and, if chronic, can result in tumorigenesis, then parotid cells possess protective mechanisms through which they can regulate resident radicles. To do so, peroxisomes harbor six enzymes with antioxidant function, namely peroxiredoxin 1 (PRDX1), glutathione peroxidase (GPX), superoxide dismutase 1 (SOD1), glutathione S transferase (GTS1), GTS-kappa and catalase. This also includes other cytoplasmic enzymes such as thioredoxin reductase 1 (TRX1) and heme oxygenase 1 (HO-1). It is thus conceivable that higher expression levels of the aforementioned antioxidant enzymes in parotid tumor tissue are either adaptation or selection to chronic redox imbalance, such that secondary high levels of antioxidant enzymes promote survival of tumor cells or precipitate apoptosis when targeted by a pro-oxidant chemotherapeutic [24,54,55]. Such findings further support the potential role of peroxisomes in cancer through their β-oxidation of fatty acids and offer perspective therapeutic targets. 

Furthermore, an active area of research is examining the role of peroxisome proliferator-activated receptors (PPARs) in cancer. PPARs are ligand-activated transcription factors that belong to the nuclear hormone-receptor family and are activated by fatty acid and lipid ligands [56]. Accumulating evidence shows that long-term activation of PPARα induces hepatocellular carcinoma in rodent, but not human, liver, suggesting that disorders in lipid metabolisms can be implicated in carcinogenesis. Conversely, PPARγ was over expressed and localized to the cytoplasm in salivary gland duct cancer suggesting it as a potential therapeutic target [57]. In fact, parotid tumor cells showed overexpression of all PPAR family members, which was inconsistent with their immunohistochemistry (IHC) staining. We thus believe that even though PPARβ and PPARγ were shown previously to have anti-proliferative, pro-apoptotic effects, their implication with regards to cancer is still unclear and warrants further investigation [56,58]. 

## 4. Materials and Methods

All subjects gave their informed consent for inclusion before they participated in the study. This study was conducted in accordance with the 1964 Declaration of Helsinki, its latest amendments and in concordance with our institutional and national research committee standards. The Ethics Committee of Justus Liebig University Giessen approved the study protocol (AZ 95/15, 25 June 2015).

### 4.1. Tissue Harvesting and Preparation

After obtaining informed consent from all participants, fresh human tissue samples were obtained following elective surgical resection of five parotid gland tumors from five patients. A healthy parotid tissue sample from each patient was included among the excised tumor mass. All Parotid tissue specimens were then immediately cryopreserved in liquid nitrogen at −80 °C for storage and transferred to our laboratory for subsequent tissue processing. One portion of our parotid specimens was processed for routine staining by hematoxylin and eosin (HE), immunofluorescence (IF), as well as Alcian blue (AB) and periodic acid Schiff (PAS) staining, while the other portion was designated for RNA extractions for detection and quantification of gene expression level.

### 4.2. Paraffin Embedding and Antigen Retrieval

Parotid tissue specimens, containing both healthy and tumor tissue, were transferred to 4% PFA in PBS at a pH of 7.4 and kept at 4 °C overnight. On the next day, Parotid tissue specimens were then embedded in paraffin (Paraplast Plus, St. Louis, MO, USA) using a Leica TP1020 embedding machine (3× 70%, 80%, 90%, 100% alcohol—90 min each; 2× xylene—90 min each; 2× paraffin—120 min each). Paraffin-embedded parotid specimens were sectioned and cut into 2, 3 and 5 µm sections with a rotation microtome (Leica SM 2000 R, Leica Instruments Nussloch, Germany) and applied on SuperFrost Plus (+) slides (Shandon, Frankfurt, Germany). Paraffin embedding, tissue sectioning and subsequent IF staining (discussed below) were carried out according to our previous publication [59,60]. Paraffin-embedded parotid sections were then immersed in xylene solvent and incubated over 3 min for deparaffinization, followed by rehydration in a series of ethanol (2× 99%, 96%, 80%, 70%, 50%, 2× aqua dest.—3 min each). Subsequently 0.01% trypsin was applied for 10 min at 37 °C to improve antigen retrieval and accessibility of epitopes. After washing in PBS, parotid sections were then put in citrate buffer (pH 6) for 3× 5 min in a microwave (600 W) followed by gradual cooling to room temperature (RT). A 4% bovine serum albumin in PBST was then used as a blocking solution for two hours. 

### 4.3. Hematoxylin and Eosin (HE) Staining

To identify the general morphology, we used a regular hematoxylin and eosin (HE) staining. Alcian blue and periodic acid Schiff (PAS) staining were performed to detect parotid secretory protein (PSP), a human salivary protein that is expressed in acinar and ductal cells of the parotid gland, submandibular gland and in gingival epithelial cells. PSP staining allows us to distinguish parotid glandular tissue from surrounding non-glandular stromal cells within our tissue sections [34].

### 4.4. Immunofluorescence (IF) Staining

After establishing the general morphology of parotid tissue sections, we aimed at distinguishing our parotid tumor samples using known parotid tumor markers. We then aimed at investigating the distribution, numerical abundance and protein composition of the peroxisomal compartment in parotid gland tumor entities in comparison to the healthy tissue. For this purpose, we used a wide array of primary antibodies against peroxisomal, mitochondrial and cytoskeleton proteins (Table 1). The validity and specificity of our chosen antibodies against peroxisomal membrane and matrix proteins have been previously confirmed in the parotid gland [34] and other human tissue organs [59,60,61,62]. Following an overnight incubation of those antibodies with our antigen-retrieved parotid sections, sections were washed three times to remove unbound primary antibodies. Next, commercially available fluorescently labeled secondary antibodies were used: anti-rabbit IgG Alexa Fluor 488 or anti-mouse Texas Red (Table 1) were subsequently added for visualization as previously described. Parotid sections without primary antibodies were incubated in parallel and served as our negative control. Finally, we counterstained the nuclear structure of our parotid cells using TOTO-3 iodide and DAPI diluted in PBS, in which blue-fluorescence stands out in vivid contrast to green, yellow or red fluorescent probes of other cellular structures in our parotid cells. Afterwards, all sections were covered with Mowiol 4.88 and N-propyl gallate in a ratio of 3:1. A Leica TCS SP5 confocal laser scanning microscope with a 63× objective and “Airy 1” setting was used for examinations and image acquisition of all our histopathological parotid gland sections.

### 4.5. RNA Extraction and Isolation

Using TissueLyser LT (Qiagen, Hilden, Germany), stored parotid tissue sections were homogenized to comparatively analyze the peroxisomal compartment in both healthy and neoplastic cells. An RNeasy Mini Kit (Qiagen, Hilden, Germany) was then used for purpose of RNA isolation and extraction. We adopted the manufacturer’s instructions previously proven to yield a high-quality RNA extract [34]. Finally, we used Agilent 2100 Bioanalyzer system and the Agilent RNA 6000 Nano Kit (Agilent Technologies, Santa Clara, CA, USA) to verify the quality and concentration of our RNA extract.

### 4.6. Quantitative Reverse Transcriptase Polymerase Chain Reaction (qPCR)

In order to obtain a gene-encoding DNA copy, the quantitative reverse transcriptase polymerase chain reaction (qRT-PCR) was adopted. We used the high-capacity RNA-to-cDNA Kit (Applied Biosystems, Weiterstadt, Germany) and the C1000 Thermal Cycler PCR system (BioRad, Dreieich, Germany). First, primers were designed using the PrimerQuest Tool (http://eu.idtdna.com/Primerquest/Home/Index, accessed date: 3 June 2018), and manufactured by Eurofins MWG Operon (Table 2). We started with a primer concentration of 5 pmol/μL, and qRT-PCR was conducted using the SYBR Select Master Mix Kit (Life Technologies, Carlsbad, California, United States of America) and the StepOnePlus Real-Time PCR System (Life Technologies, Carlsbad, California, United States of America). The following operational standards were used: denaturation at 94 °C for 4 min; 45 cycles of denaturation at 95 °C for 15 s; annealing at 60 °C for 60 s, and extension at 7 °C for 1 min. As a final step, RT-values were divided by housekeeper HPRT values in order to normalize for different mRNAs. The RNA of three different human healthy and tumor parotid tissues were used in duplicates.

### 4.7. Statistical Methods and Tools 

To calculate the statistical significances, we used the unpaired t-test with the Graphpad Prism software, version 6.01. Fold changes were analyzed by an approximation method taking the ΔΔCt values into consideration. 

## 5. Conclusions

We demonstrated the presence of differential expression of peroxisomal proteins between healthy and tumor tissue of the human parotid salivary gland. Upregulation of biosynthesis alongside downregulation of antioxidant key enzymes suggests that peroxisomes in parotid gland tumors have a pro-tumorigenic role. Such a role appears variable between subtypes of parotid tumors. Our findings supplement current research in the field and add perspectives on the effectiveness of peroxisomes as potential molecular targets and or predictive biological markers. 

## Figures and Tables

**Table 1:** List of antibodies used for the detection of peroxisomal proteins in the parotid gland.

Primary Antibody	Host	Target Molecular Weight	Dilution (IF)	Dilution (WB)	Supplier
**Cell type specific antigens**					
Parotid secretory Protein, mouse (SPLUNC2)	Goat, polyclonal	25 kDa	-	1:3000	Acris Antibodies, Inc. Cat. no.: AP31965PU-N
Parotid secretory Protein, human (SPLUNC2)	Mouse, monoclonal	25 kDa		1:1000	Abbexa, Cat. no.: abx11413
**Peroxisomal biogenesis and metabolic proteins**					
Peroxin 6 (PEX6p),	Rabbit, polyclonal	104 kDa		1:1000	Novus Biologicals, cat. no.: NBP1-80955
Peroxin 7 (PEX7p),	Mouse, monoclonal	40 kDa		1:1000	UC Davis/NIH NeuroMab Facility
Peroxin 13 (PEX13p), mouse	Rabbit, polyclonal	44 kDa	1:1000	1:6000	Gift from Denis I. Crane; School of Biomol. Biophys. Sci., Griffith Univ., Nathan, Brisbane, Australia; see reference: [63]
Peroxin 14 (PEX14p), mouse	Rabbit, polyclonal	42 kDa	1:1000	1:3000	Gift from Denis I. Crane; see reference: [63]
Catalase (CAT), mouse	Rabbit, polyclonal	60 kDa	1:2000	1:5000	Gift from Denis I. Crane, see reference: [63]
ABC-transporter D3 (ABCD3/PMP70), mouse	Sheep, polyclonal	75 kDa	1:1000	1:3000	Gift from Stephen Gould, Johns Hopkins Univ., Dept. Biol. Chem., Baltimore, MD, USA; see reference: [64]
Acyl-CoA oxidase (ACOX 1), mouse	Rabbit, polyclonal	51 kDa		1:5000	Gift from Paul P. van Veldhoven, Dept. of Molecular Cell Biology, Pharmacology, Catholic University Leuven, Belgium; see reference: [65]
Peroxiredoxin 1	Rabbit, polyclonal	22 kDa		1:1000	Abcam, Cambridge, UK, Cat. no: ab59538
“SKL”, mouse peptide	Rabbit, polyclonal			1:5000	Gift from Denis I. Crane, see reference: [63]
Thiolase	Rabbit, polyclonal	51 kDa	1:1000	1:5000	Gift from Nancy E Bravermann; Depts. of Human Genetics and Pediatrics, McGill University-Montreal Montreal, QC, Canada.
Alkylglycerone-phosphate synthase (AGPS)	Mouse, monoclonal	78 kDa	1:1000	1:500	Santa cruz, Cat no: sc-374201
Glyceronephosphate O-Acyltransferase(GNPAT)	Rabbit, polyclonal	70 kDa	1:500	1:5000	Proteintech, Cat no: 14931-1-AP
**Nuclear receptors and cell signalling molecules**					
PPAR α, rabbit	Rabbit, polyclonal	52 kDa		1:1000	Santa cruz, Cat no: sc-9000
PPAR α	Mouse, monoclonal	52 kDa		1:1000	Millipore, Cat. no.: MAB3890
PPAR α	Mouse, monoclonal	52 kDa		1:1000	Pierce Biotechnology, Cat. no.: MA 1--822
PPAR β, rabbit	Rabbit, polyclonal	52 kDa	1:50	1:1000	Santa cruz, Cat no: sc-7197
PPAR δ	Rabbit, polyclonal	50 kDa		1:1000	Abiocode, Cat. no.: R2295-1
PPAR γ, rabbit	Rabbit, polyclonal	55 kDa	1:50	1:1000	Santa cruz, Cat no: sc-7196
**Antioxidative enzymes from other cell compartments**					
Glutathione reductase	Rabbit, polyclonal	56 kDa		1:1000	Abcam, Cambridge, UK, Cat. no: ab16801
Oxidative phosphorylation complex III (OxPhosIII), human	Mouse, monoclonal			1:1000	Molecular Probes/Invitrogen, Carlsbad, USA, Cat. no.: A11143
Thioredoxin-1 (TrxR1)	Mouse, monoclonal	55 kDa		1:1000	Santa cruz, Cat no: sc-28321
Superoxide dismutase 1 (SOD-1),	Goat; polyclonal	17 kDa		1:5000	R&D Systems, Cat. no.; AF3787
Superoxide dismutase 2 (SOD-2)	Rabbit, polyclonal	25 kDa	1:1000	1:1000	Research diagnostics, Inc., NJ, USA, Cat no: RDI-RTSODMabR
HO-1	Rabbit, polyclonal	32 kDa	1:1000	1:1000	Stressgen, Cat no: SPA-895
**Other marker proteins of different cell compartments**					
β-tubulin,	Mouse, monoclonal	55 kDa		1:10,000	Sigma Aldrich, Inc., Cat. no.: T8328
β-actin	Mouse, monoclonal	45 kDa		1:10,000	Cell Signaling Technology, Inc.#3700
Glyceraldehyde-3-phosphate dehydrogenase (GAPDH)	Mouse, monoclonal	36 kDa		1:60,000	Hy Test Ltd., Cat. no.:5G4
Secondary Antibodies					
HRP-rabbit				1:6000	Cell Signaling Technology, Inc.#
HRP-mouse				1:6000	Cell Signaling Technology, Inc.#
Bovine anti goat HRP				1:5000	Santa cruz, Cat. no: sc-2378

**Table 2:** List of the human primers used for qPCR analyses.

Gene Target	Gene Bank Accession No.	Sence Primer (5′-3′)	Antisense Primer (5′-3′)	Annealing Temp °C	PCR Product (bp)
*ABCD1*	NM_000033.3	GTGGAGGACATGCAAAGGAA	TCACACATAGCCTCCCAACC	58.1	113
*ABCD3*	NM_002858.3	ATGACCCTTGGAACACTTCG	TGCCATCCATATGCAGGTAG	57.8	385
*ACOX1*	NM_004035.6	ATTTCCTTCAGGGGAGCATC	GCCAAGTGTCACATCCTGAA	57.3	137
*ACOX2*	NM_003500.3	CAAATTGTCGGCCTCCTGTA	GAGATCTCTGTGGCGTGGAG	57.9	125
*ACOX3*	NM_003501.2	GGAGTGTGTGGGCTCTTATC	CTCTTGCTCGGTAGGCATC	57.7	107
*ACTB*	NM_001101.3	TCCCTGGAGAAGAGCTACGA	AGCACTGTGTTGGCGTACAG	59.4	194
*AGPS*	NM_003659.3	AGGGGGATCGTGAGAAGGT	CCAAAGCCAAGTCTCGAATG	59.6	147
*CAT*	NM_001752.3	CGTGCTGAATGAGGAACAGA	TTGTCCAGAAGAGCCTGGAT	57.9	150
*FDPS*	NM_001135821.1	CAAGGAGGTCCTGGAGTACAA	GGAGACTATCAGCATCCTGTTTC	58.7	113
*GAPDH*	NM_002046.5	GTCAACGGATTTGGTCGTATT	TGTAGTTGAGGTCAATGAAGGG	56.6	106
*GNPAT*	NM_014236.3	GTGCAGAAAAACGCCTTAGC	GGCTGGTTTTCCTATTGGTG	58.3	150
*GPX1*	NM_000581.2	CAGTTTGGGCATCAGGAGAA	TCGAAGAGCATGAAGTTGGG	57.8	101
*GR1/GSR*	NM_000637.3	GTGGCCTCCTATGACTACCT	CATCCAACATTCACGCAAGTG	57.9	137
*HMGCR*	NM_000859.2	CGATGCATAGCCATCCTGTAT	GCTGGAATGACAGCTTCACA	57.7	87
*HMGCS*	NM_001098272.2	TCTATCCTTCACACAGCTCTTTC	GGCAACAATTCCCACATCTTT	57.9	89
*HO-1*	NM_002133.2	CGGCTTCAAGCTGGTGAT	AGCTCTTCTGGGAAGTAGACA	57.7	114
*HPRT*	NM_000194.2	CACTGGCAAAACAATGCAGACT	GTCTGGCTTATATCCAACACTTCGT	60.2	118
*IDI*	NM_004969.3	TCTCATTGGGCATGAAGGTC	CATAAAACCTCGGGCTCCTT	57.6	106
*MFP1*	NM_001966.3	ATGGATATGGATGGCCAAGG	GCTCCAGTTGGGGAATATCA	57.1	126
*MFP2*	NM_000414.3	TGTCGTTGCAGGCCTTATT	CCTCCCAAATCATTCACAACAAC	57.4	148
*MVD*	NM_002461.2	GGTGGCACCTGTTCTTCTCTCT	CTGATGAGCAGCTGTCTGGAGT	56.5	82
*MVK*	NM_000431.3	CTGGACACAAGCTTTCTGGA	AAGCCTGCAACCTCCTTTAG	57.7	83
*PMVK*	NM_006556.3	GCCTTTCGGAAGGACATGAT	GTCACTCACCAGCCAGATG	58	114
*PPARalpha*	NM_005036.4	CTGGCCAAGAGAATCTACGAG	ACTGGTTCCATGTTGCCAAG	57.9	
*PPARbeta*	NM_177435.2	AACATGCAAGGCACTGACTG	CTGCCAAAGTGCTGGGATT	59	129
*PPARgamma*	NM_138712.3	ATCTTTCAGGGCTGCCAGT	TCGTGGACTCCATATTTGAGG	58.9	131
*PRDX6*	NM_004905.2	TTAGTGCCATGTGCCTTTCA	TAGCAACCCACTGCAAGAAG	57.7	144
*MVD*	NM_002461.2	GGTGGCACCTGTTCTTCTCTCT	CTGATGAGCAGCTGTCTGGAGT	56.5	82
*MVK*	NM_000431.3	CTGGACACAAGCTTTCTGGA	AAGCCTGCAACCTCCTTTAG	57.7	83
*PMVK*	NM_006556.3	GCCTTTCGGAAGGACATGAT	GTCACTCACCAGCCAGATG	58	114
*PPARalpha*	NM_005036.4	CTGGCCAAGAGAATCTACGAG	ACTGGTTCCATGTTGCCAAG	57.9	
*PPARbeta*	NM_177435.2	AACATGCAAGGCACTGACTG	CTGCCAAAGTGCTGGGATT	59	129
*PPARgamma*	NM_138712.3	ATCTTTCAGGGCTGCCAGT	TCGTGGACTCCATATTTGAGG	58.9	131
*PRDX6*	NM_004905.2	TTAGTGCCATGTGCCTTTCA	TAGCAACCCACTGCAAGAAG	57.7	144
*PSP/SPLUNC2*	NM_001319164.1	GAAGTCTGAGGTGGTGTCAAG	TGCCAAGATTGTCAAGAAGAGA	58.2	107
*RPL13*	NM_000977.	CGGAATGGCATGGTCTTGA	CCTTACGTCTGCGGATCTTAC	57.8	100
*SOD1*	NM_000454.4	AGGATGAAGAGAGGCATGTTG	ATGGTCTCCTGAGAGTGAGAT	57.7	107
*SOD2*	NM_000636.2	GTTGGCCAAGGGAGATGTTA	CGTTAGGGCTGAGGTTTGT	57.5	110
*SQS*	NM_001287742.1	GAAGTCAGTGAGACCAAGAACC	CGCTCTCTGTAGAGCCTTAGA	58.6	76
*TBP*	NM_003194.4	TGACCCAGCATCACTGTTTC	GCTGGAACTCGTCTCACTATTC	58.1	118
*Thiolase*	NM_001607.3	GATGCCTTCTTACCCCAACA	CCCAACCACTGCATAAGACC	57.5	
*TRX1*	NM_001244938.1	GGACGCTGCAGGTGATAAA	CACTCTGAAGCAACATCATGAAAG	57.9	102
*TRX2*	NM_012473.3	GTTAGAGAAGATGGTGGCCAAG	GCTGACACCTCATACTCAATGG	58.7	99

## Data Availability

The data presented in this study are contained within the article. Further supporting material is available upon request.
